# Obsidian: A Pioneering Natural Resource for Green, Fire-Resistant Composite Material Industries

**DOI:** 10.1021/acsomega.4c07737

**Published:** 2025-01-17

**Authors:** Hassan Soltan Hassan, Ahmed S. Elshimy, Isabel Israde-Alcantara, Hamdy A. Abdel-Gawwad, Heriberto Pfeiffer

**Affiliations:** † Instituto de Investigaciones en Materiales, Universidad Nacional Autónoma de México, Circuito exterior s/n, Ciudad Universitaria, Del. Coyoacán, Ciudad de México 04510, Mexico; ‡ Geology Department, Faculty of Science, New Valley University, El- Kharga 72511, Egypt; § Faculty of Earth Science, Beni−Suef University, Beni−Suef 62511, Egypt; ∥ Instituto de Investigaciones en Ciencias de la Tierra, Universidad Michoacana San Nicolás de Hidalgo, Morelia, Michoacán 58030, México; ⊥ Raw Building Materials Research and Processing Technology Institute, Housing and Building National Research Center (HBRC), Cairo 12311, Egypt

## Abstract

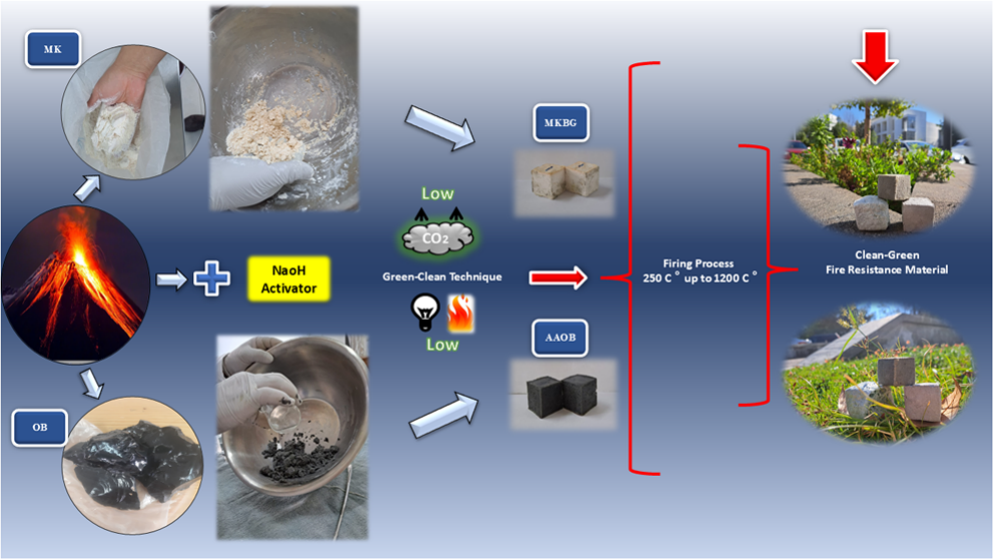

A cleaner production approach was employed to develop an innovative and eco-friendly, fire-resistant composite material that boasts exceptional mechanical performance and is capable of withstanding harsh conditions. Novel obsidian (OB) and metakaolin (MK) were individually mixed with NaOH at different ratios ranging from 8 to 12 wt %. Each material was subjected to only 80 °C, forming alkali-activated obsidian (AAOB) and metakaolin-based geopolymer (MKBG). Extensive analyses, including compressive strength, thermal conductivity, X-ray diffraction (XRD), X-ray fluorescence (XRF), thermogravimetric analysis (TGA), Fourier transform infrared (FTIR) spectroscopy, scanning electron microscopy (SEM), and energy-dispersive X-ray (EDX) analysis, confirmed the performance of both AAOB and MKBG. The AAOB outshined MKBG in fire resistance and mechanical strength, boasting impressive compressive strengths of 36.5, 69, and 101 MPa, respectively at day one. In contrast, MKBG lagged behind, with compressive strengths of 9.1, 23.24, and 25.66 MPa under the same conditions. Furthermore, AAOB exhibited a significantly higher porosity (80%) at 1000 °C and a lower thermal conductivity of 0.193 W/mK, compared to MKBG, which possessed a lower porosity (33%) and higher thermal conductivity of 0.901 W/mK. The AAOB represents a significant leap in the green revolution for sustainable fire-resistant composite materials. Its versatility extends across various sectors, notably in ultrahigh-temperature industrial and construction applications.

## 1 Introduction

In the vast expanse of the Azufres Caldera, a type of volcanic landscape spanning an impressive 30 km and rising to an elevation of 3500 m above sea level in west-central Mexico, two primary natural resources stand out: Obsidian (OB) and kaolinitic clays (KC).^1−3^ Obsidian is characterized by its rigid, glassy composition with a brittle and amorphous texture, featuring razor-sharp edges due to the rapid cooling of silica-rich felsic igneous rocks (making up 50–80% of its composition). This volcanic glass is abundant and significant within the Azufres Caldera.^1−3^

Several regions in Mexico are home to deposits of volcanic glass known as obsidian, which is formed from rhyolitic magmatic activity. These silica-rich deposits (containing 65–80% silica) and alumina flow at temperatures around 600 °C, cooling so rapidly that they do not have time to crystallize, resulting in an amorphous, glassy material.^1−3^ Obsidian is naturally brittle and has a hardness of 5–6 on the Mohs scale. Extensive outcrops of obsidian can be found in northwestern Mexico, specifically in Durango, Nayarit, Zacatecas, and Sonora. In central Mexico, significant deposits are located in Puebla, Guanajuato, Hidalgo, Michoacán, and Jalisco, which are home to the third-largest obsidian deposits in the world. The formation of these glassy rocks varies in Jalisco; they often break into columnar shapes, while in Michoacán, they appear as irregular masses. Some of the pure black obsidian deposits are located near the Los Azufres Caldera geothermal field in northeastern Michoacán. However, there are many color variations of obsidian, with red and green being common.^1−3^

Historically, several well-known obsidian deposits were extensively utilized by the Aztec Empire and the Tarascan state, both of which flourished between 1300 and 1521. They valued obsidian for its sharpness and durability, using it to craft weapons, tools such as knives and spearpoints, scrapers, and arrowheads, as well as adornments. Obsidian was also a key trade commodity, often exchanged for items like salt, jade, and malachite.^1−3^

Kaolin is a type of clayey rock primarily composed of the mineral kaolinite, with the chemical formula Al_2_(Si_2_O_5_) (OH)_4_. Kaolinite is recognized for its nearly pure white color, fine particle size (approximately 1–2 μm), nontoxic nature, low abrasiveness, and chemical stability. These qualities make kaolin a highly versatile mineral that is used across a broad range of industries. However, the presence of impurities, particularly iron oxides, can affect its potential applications by causing color variations in the finished products.^4^ The growing demand for nanocomposite materials, such as those used in printer inks and in the engineering and medical fields, has spurred increased scientific and economic interest in kaolin. Through a process known as activation, kaolinic clay can be transformed into metakaolin (MK). This process involves heating the original kaolin rock, which gives the clay a powdery texture and a creamy-white color. Kaolin’s high alumina and silica content is key to its transformation into metakaolin, as these properties are enhanced through calcination.^5^

In Mexico, kaolin deposits are widely distributed, with major deposits located in states such as Chihuahua, Guanajuato, Veracruz, the Azufres Caldera region in Michoacán, and Hidalgo. These deposits are typically found along the Trans-Mexican Volcanic Belt (TMVB). Despite the extensive presence of these deposits, local production does not fully meet the nation’s requirements, leading to the need for imported kaolin.^4,5^ Significant kaolin deposits are also found in the eastern part of the TMVB, especially in the states of Puebla, Hidalgo, and Veracruz. In Puebla, kaolin deposits are located in the Acoculco area, a volcanic caldera complex with geothermal potential mainly composed of acidic volcanic rocks that have undergone widespread hydrothermal alteration. Kaolinite found in this region is associated with minerals, such as alunite, opal, tridymite, and anatase, resulting from the advanced argillic alteration of tuffs and volcanic breccias. Though the Acoculco deposits have been intermittently exploited, they have not been the subject of thorough studies.^4,5^

In the vicinity of Acoculco, two other notable kaolin deposits, Agua Blanca in Hidalgo and Huayacocotla in Veracruz, hold economic significance. The Agua Blanca deposit has been investigated in the past and is currently being mined. The Huayacocotla deposit, which has been actively mined since 1952, serves as the main economic driver for the municipality bearing the same name.^4,5^

Recent studies^6,7^ have spurred researchers to explore alternative building materials as a means to mitigate the detrimental impact of global carbon emissions and reduce energy consumption. To illustrate, it is estimated that the production of ordinary portland cement (OPC) results in approximately 1.5 billion tons of CO_2_ emissions annually, accounting for roughly 5% of the total atmospheric CO_2_^8,9^ An intriguing prospect lies in the utilization of alkali-activated and geopolymeric materials, which both demonstrated remarkable mechanical and thermal properties.^6,7,10^ In 1972, Joseph Davidovits introduced the term “geopolymers,” defining them as inorganic polymer materials that arise from the reaction between alkaline solutions (such as sodium hydroxide, potassium hydroxide, sodium silicate, and potassium silicate) and an aluminosilicate substance like fly ash, ground granulated blast-furnace slag, or metakaolin (MK).^11^

In the contemporary landscape, the use of geopolymers at elevated temperatures stands out as notable and propitious for their practical deployment. This encompasses diverse existing products within the market, such as the innovative Nu-Core A2FR characterized by its geopolymer composite panels, the cutting-edge Ino-Flamm boasting fire-resistant geopolymer paint, and the resourceful SKOBIFIX 30 presenting a geopolymer foam exclusively designed for heating systems.^12^ In the geopolymers case, especially in those based on metakaolin and fly ash, relatively large works connected with the behavior of these materials at elevated temperatures have been conducted.^13,14^ These investigations include fire resistance, as well as temperature resistance of these materials, and the influence of temperature on mechanical properties, among others.^15−17^ The results obtained show different behaviors for these types of materials; while some researchers report a decrease in the mechanical properties of materials with temperature,^18^ others report an increase. For example, Shaikh and Haque^17^ noticed an increase in the mechanical properties of geopolymer composites reinforced by fibers at temperatures of 200, 400, and 600 °C compared with the materials tested at an ambient temperature of 28 °C, but decreasing the values at 800 °C.

The utilization of cement-based porous materials as inorganic thermal insulations is common. However, a notable drawback of this method is the susceptibility of such materials to spalling and, in specific conditions, explosively spalling when subjected to high temperatures. This trait raises significant concerns, particularly in the event of accidental fires, as it could potentially result in catastrophic consequences such as the complete collapse of the building.^15,19^ Additionally, traditional cement manufacturing is considered as a risk to the environment because it produces up to 8% of the world’s CO_2_ emissions.^20^ Geopolymer foams and alkali-activated porous materials emerge as promising alternatives to enhance fire resistance and mitigate the significant drawbacks associated with cement-based porous materials used in inorganic thermal insulation^21,22^ These materials are collectively referred to as geopolymer foams. Recent studies have delved into exploring the effectiveness of geopolymer foams in addressing the limitations of traditional cement-based materials. The findings indicate that geopolymer foams can substantially improve fire resistance performance while maintaining environmental friendliness.^21,22^ This approach not only lessens the adverse environmental impacts but also enhances the fire-resistant capabilities of the material.^15,23,24^

As the world grapples with a global energy crisis, there has been a growing focus on building energy conservation. Thermal insulation materials, which play a crucial role in minimizing heat flow or heat loss through building envelopes, have gained significant attention in this context.^25,26^ However, common materials such as polystyrene board, polystyrene foams, and polyurethane foams, despite their effectiveness in insulation, have been associated with various building fire tragedies due to their flammability and the emission of poisonous gases.^27,28^ In response to these concerns, scientists are now exploring the potential of inorganic porous materials with low thermal conductivity, nonflammability, and nontoxic qualities. These materials show promise as alternatives that could enhance building fire protection.^29,30^

The assessment of OB rock as a primary precursor for producing durable, fire-resistant material through the alkali-activation process has not been explored until now. The novelty of this current study lies in synthesizing a clean green substance capable of withstanding high temperatures and extreme conditions. This process involves using natural volcanic OB, abundant in the Mexican region, without the necessity for any primary thermochemical treatment. Unlike traditional thermal insulators, this state-of-the-art material has been developed through a simple, environmentally friendly approach, resulting in sustainable solutions. AAOB cementitious insulators provide an alternative approach to addressing the challenges of high-cost and fire-resistant materials by leveraging highly accessible natural resources to create a novel and cost-effective thermal insulator that is more efficient and applicable compared with MKBG insulators. The final products of AAOB are anticipated to be both safe and environmentally conscious, offering a promising substitute for existing thermal insulators that require high energy consumption and release significant amounts of CO_2_ and other greenhouse gases.

## 2 Experimental Study

### 2.1 Resources and Characterization of Materials

The initial raw materials employed in this study were two natural resources: OB and KC. Unique black bright Mexican OB samples originating from Pleistocene volcanic deposits in the Los Azufres zone, Michoacán state, Mexico, were collected. Meanwhile, KC was sourced from the same region but from a distinct deposit located 8 km away. MK was produced by subjecting kaolinitic clay to thermal treatment at 800 °C for 2 h, with a heating rate of 5 °C/min.

**Figure 1:**
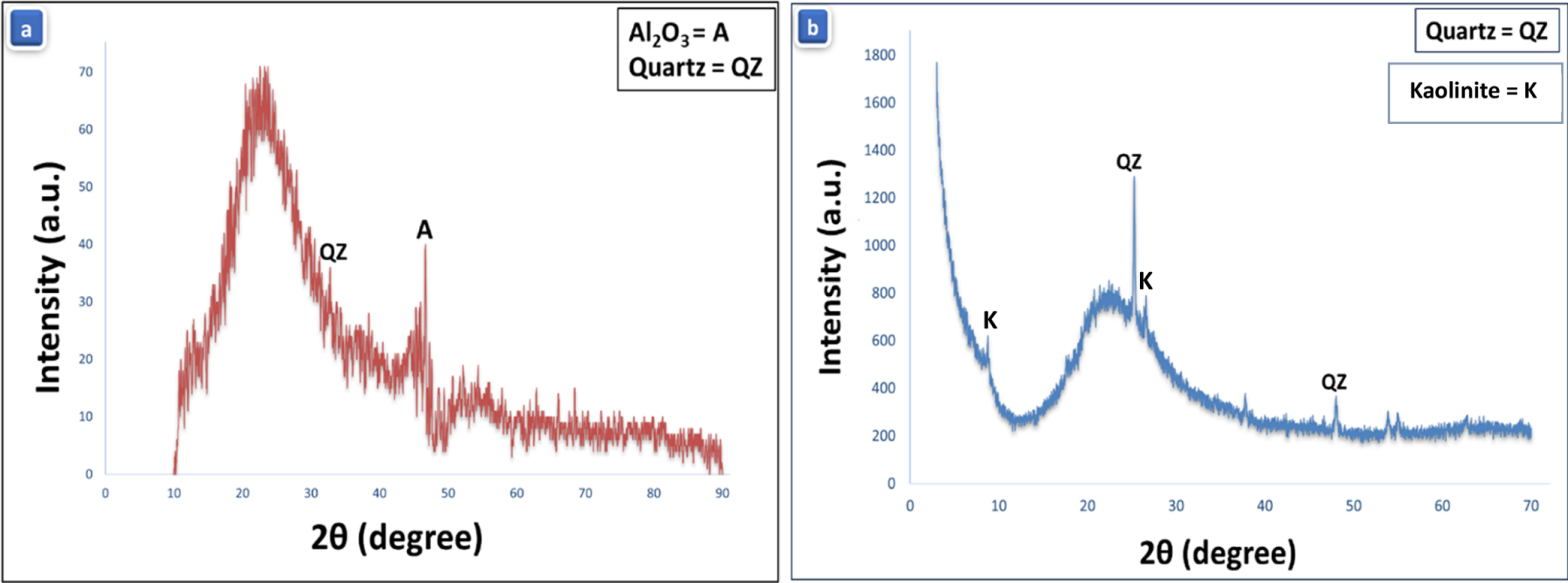
XRD patterns of OB (a) and MK (b) precursors.

The chemical compositions of both materials are listed in Table 1. It is indicated that OB had a high silica content and a moderate amount of alumina, whereas MK is distinguished by high Si and Al contents. Sodium hydroxide (NaOH, 99.99%, from Fisher Scientific Chemical Company, U.K.) was the only alkaline activator used. As proved by X-ray diffraction (XRD) (Figure 1a), OB has a vitreous structure with a hump in a 2θ range of 10 to 35°, where the Quartz crystal peak appears at 31° and Al_2_O_3_ at 47° 2θ, respectively. MK also exhibits an amorphous pattern with the appearance of the crystalline peaks affiliated to Quartz at 26° and Kaolinite at 9 and 28° 2θ, respectively (Figure 1b). Regarding the physical properties of both starting materials, MK recorded a specific gravity of 2.30, while OB demonstrated a higher value (2.60).^1−3,31^

**Table 1:** Chemical Compositions (wt %) of the Starting Materials

component	SiO_2_	CaO	MgO	Fe_2_O_3_	Al_2_O_3_	SO_3_	CL	Na_2_O	K_2_O
Obsidian	59.46	6.69	3.87	2.86	15.73	2.23	1.55	3.22	3.33
metakaolin	53.1	0.30	1.22	0.77	41.40	0.22	1.20	0.33	0.25

### 2.2 Green Synthesis of AAOB and MKBG Samples

A natural volcanic sample comprising OB and MK underwent the grinding process using a vibrating milling machine, ultimately passing through a 75 mm sieve. This yielded a finely powdered concoction from both OB and MK, exhibiting homogeneity. The alkali-activation process of OB and MK led to the formulation of six distinct mixtures. Varied concentrations of NaOH (as detailed in Table 2) were employed separately in this activation process. Activation was achieved through different weight percentages of Na_2_O (as NaOH), namely, 8, 10, and 12 wt %. The rationale behind this diversity was to assess how these concentrations influence the performance of the treated powders. To maintain optimal conditions, the NaOH was allowed ample time to cool before utilization, mitigating the risk of exothermic reactions that could potentially impact the workability of the prepared pastes. The water content in the mixture is primarily contingent on the aluminosilicate source. Notably, OB exhibited a lower water content compared to MK. Specifically, the water-to-powder ratios (W/P) required for crafting workable pastes ranged from 0.25 to 0.28 for OB and 0.43 to 0.45 for MK, respectively.

**Table 2:** Compositions of Different OB and MK Mixtures Containing NaOH for Sample Preparation

	OB	MK	Na_2_O (as NaOH)	
mixtures notations	wt %			W/P ratio
OB1	100		8	0.25
OB2	100		10	0.26
OB3	100		12	0.28
MK1		100	8	0.43
MK2		100	10	0.44
MK3		100	12	0.45

Upon introduction of NaOH into the aluminosilicate powder, a deliberate mixing protocol was followed. Initially, a slow mixing rate of 80 rpm was applied for a duration of 3 min. Subsequently, a fitting mixing rate was employed for an additional 3 min to achieve thorough homogeneity in the mixture. The freshly prepared pastes were then carefully transferred into stainless steel molds measuring 50 × 50 × 50 mm^3^. To ensure the removal of any potential air bubbles that might have formed during the mixing process, the molds were subjected to vibration for 10 s within a specialized vibrating table. Following this step, plastic sheets were promptly employed to cover the molds, preventing moisture loss during the crucial setting process. The molds, now housing the freshly prepared pastes, were collectively placed in an oven set at 80 °C for a duration of 24 h. After 24 h and once the AAOB and MKBG had solidified, they were demolded into samples with dimensions of 1 in. each. These samples were then allowed to cure in the laboratory at ambient temperature until the designated testing period. The various tests, notably the compressive strength tests, were subsequently conducted.

### 2.3 Processing of Fire Exposure Test

**Figure 2:**
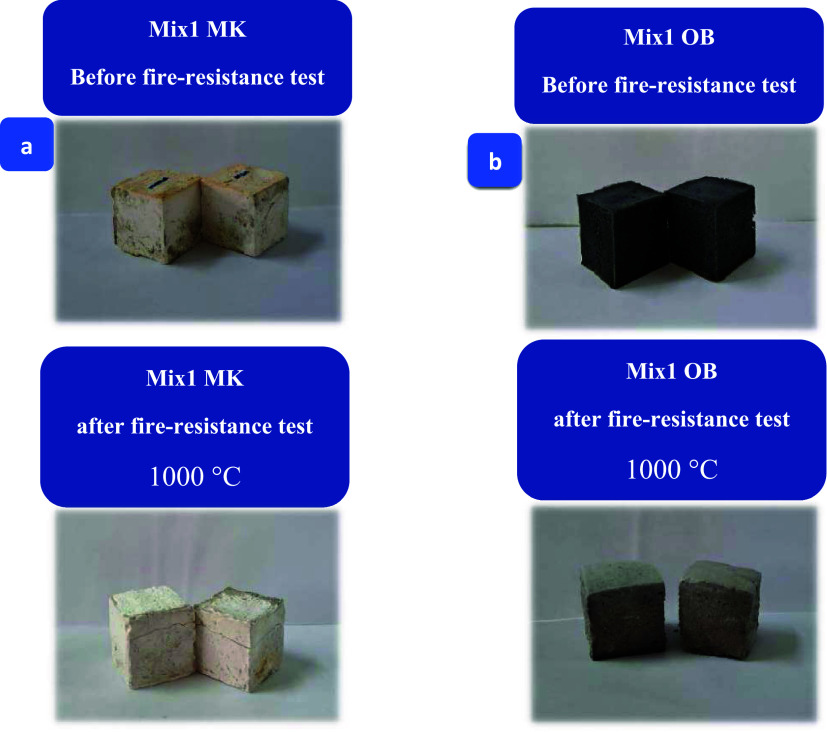
Digital photos obtained before (a) and after (b) the process of the fire resistance test to MK and OB specimens.

The fire resistance tests were conducted following the ASTM E119 standard.^32^ However, the focus of this study was to assess the performance of the AAOB and MKBG specimens under different aggressive environmental conditions. To simulate this scenario, a heating rate of 15 °C/min was employed. This accelerated heating rate was used to evaluate the specimens’ ability to withstand the intense heat generated during aggressive fire exposure. Three samples of each combination were heated at elevated temperatures (250, 550, 750, 1000, and 1200 °C) into a furnace for 2 h of curing. The specimens were held at a temperature for a total of 2 h after achieving each target temperature in order to homogenize the temperature throughout the specimens. The specimens were gradually cooled after being exposed to high temperatures by opening the furnace vent hole to room temperature. After being cooled, the prepared specimens were conserved in suitable desiccators. Following the fire exposure test, the specimen unite volume, bulk density, and compressive strength tests were conducted. Unveiling authentic digital images portraying both MKBG and AAOB specimens (activating at Na_2_O of 8 wt %) that cured at 1000 °C, which these images serve as tangible proof of their transformation before and after undergoing a rigorous firing resistance test. These remarkable visual color changes can be observed in Figure 2a,b.

### 2.4 Evaluation and Characterization of AAOB and MKBG Samples

#### 2.4.1 Experimental Methods

Relative volume change (*V*_c_) as a result of thermal treatment was determined by dividing the volume of the fired sample (*V*_f_) by the original volume (*V*_i_) of the same sample before firing multiplied by 100 according to eq 1
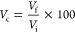
1

Compressive strength was tested on three hardened cement cubes using a five-ton German-Bruf-Pressing Machine with a loading rate of 175 kN, as per ASTM C109 M.^33^ After the evaluation, the hydration reaction was stopped by submerging the broken samples in a 1:1 volumetric mixture of methanol and acetone for 24 h, followed by 3 h of drying at 80 °C and storing the samples in a tightly sealed jar until analysis. All the above-mentioned methods were conducted on three samples of each mixture, and then the average was calculated and recorded. Finally, Archimedes’ principles provided the foundation for determining the physical characteristic known as apparent porosity, which involves immersing an object in water to assess its properties.^34^

#### 2.4.2 Instrumental Techniques

The Philips PW3050/60 diffractometer was used for X-ray diffraction (XRD) to determine both AAOB and MKBG, with a scanning range of 5–90 (2θ°), a scanning speed of 1s/step, and a resolution of 0.02/step. The quantitative content and crystallinity level were determined by using software to perform a Rietveld quantitative XRD analysis.^35^ Thermogravimetric analysis (TG) was performed using a DT-50 Thermal Analyzer. It was conducted by heating 15 mg of powdered sample (≤25 μm) from 50 to 1000 °C with a heating rate of 10 °C/min in a nitrogen environment (Shimadzu Co-Kyoto, Japan). Fourier transform Infrared (FTIR) spectroscopy was applied to identify the functional groups inside the fired and unfired samples. It was carried out using the KBr discussing Genesis-II FTIR spectrometer at a wavenumber range of 400–4000 cm^–1^.

For the analysis of microstructural development, a JEOL model JSM-7600F Schottky field emission scanning electron microscope (FE-SEM) equipped with an energy-dispersive X-ray (EDX) for elemental analysis was employed. The instrument features an energy-dispersive X-ray spectroscopy (EDS) system providing a resolution of 1.0 nm at 133 eV. To mitigate electron accumulation and ensure electrical conductivity, a fine layer of gold is applied to the sample prior to acquiring SEM images. Finally, an applied precision ISOMET 2104 was employed to measure the thermal conductivity of both the unfired and fired samples.

## 3 Results and Discussion: Findings and Insights

### 3.1 Compressive Strength

**Figure 3:**
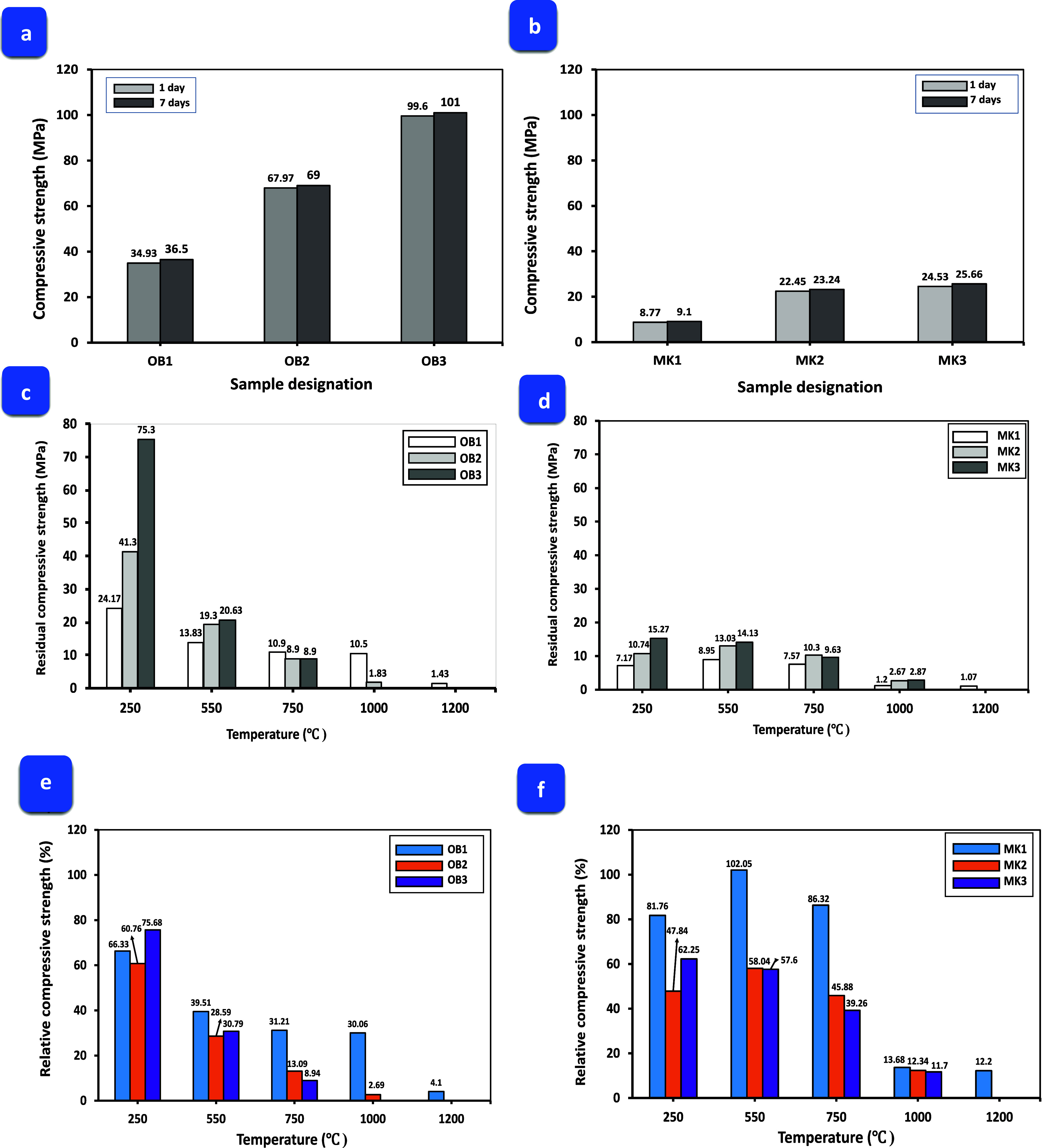
Compressive strength of AAOB and MKBG samples after 1 and 7 days (a, b), residual samples (c, d), and relative (e, f) compressive strength versus exposure temperatures for both mixtures, respectively.

The compressive strength of the (AAOB) and (MKBG) under varying curing ages ranging from 1- to 7 days was depicted in Figure 3a,b. A superior enhancement in the compressive strength of AAOB at standard conditions (dried at 80 °C) was observed upon increasing the concentration of Na_2_O-derived NaOH between 8 and 12 wt %. The hardened AAOB sample, containing 12 wt % of Na_2_O, exhibited compressive strength that was approximately 185 and 94.6% higher than those activated by 8 and 10 wt % (as Na_2_O) of the same activator, respectively. This improvement is attributed to the sustained dissolution of obsidian amorphous alumina-silicate content, leading to an increase in the formation of sodium aluminum silicate hydrate (Na–Al–S–H) phases which are responsible for the high compressive strength effect. In comparison, the activation of MK under the same physicochemical conditions demonstrates a somewhat similar performance trend. However, AAOB exhibits a compressive strength behavior higher than that of MKBG at similar alkali-activator concentrations. This is due to the higher content of amorphous silicate phases present in OB forming more strength-giving phases (Na–Al–S–H) compared to MK. Additionally, pozzolanic materials, such as MK, lead to an increase in the standard water consistency of a paste,^36^ due to their finer grain size, causing the alkaline solution to dissolve and reducing its reactivity which declines the production of the new species. Furthermore, substantial amounts of alkalis are required for the dissolving of reactive aluminate species from MK,^36^ in contrast to the OB which contains a lower alumina content. Moreover, there is only a slight increase in the compressive strength behavior of AAOB and MKBG after 7 days. This can be attributed to the fact that 1 day is sufficient for the formation of nearly all strength-enhancing phases for both activated materials.

**Figure 4:**
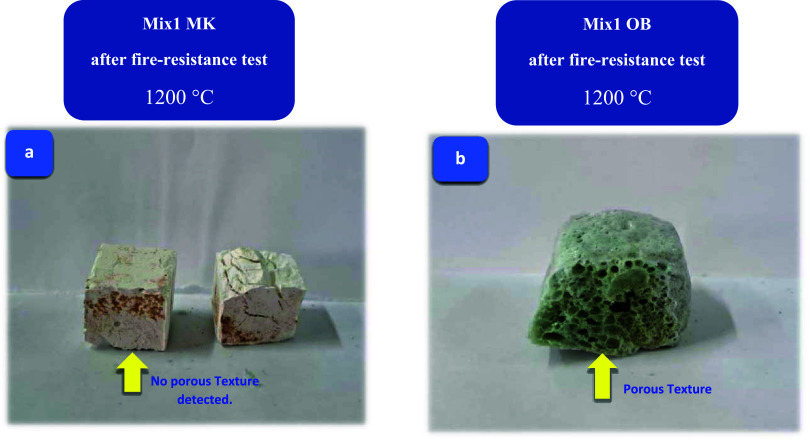
Morphological appearance at 1200 °C of the foamed (a) AAOB sample (porous structure) and (b) destroyed MKBG.

The residual and relative compressive strengths of the AAOB and MKBG mixtures were studied and plotted against exposure temperatures ranging from 250 to 1200 °C in Figure 3c–f. AAOB mixtures evince residual compressive strengths exceeding those of MKBG at temperatures of 250 and 550 °C, attributable to the heightened reactivity of OB compared to MK, culminating in the generation of larger quantities of Na–Al–Si–H. Moreover, increasing the temperature from 550 to 1200 °C leads to the crystallization of mullite and nepheline minerals for both AAOB and MKBG as proved by XRD-data, see below Figure 7a,b. Although, in the similarity in mineral composition of both AAOB and MKBG specimens, the AAOB’s first mixture (OB1) represents higher residual compressive strength than MKBG’s first one at 1200 °C. This may be ascribed to the heightened levels of mullite and nepheline present within AAOB relative to MKBG, which formed the porous structure of the AAOB mixture (Figure 4b). Therefore, OB1 was the optimal mixture as compared with all AAOB and MKBG samples (Figure 4a), which can be used as a thermal insulation material and other more advanced applications.

In contrast, the MKBG mixture showed a cracked and cohesionless geometry after being exposed to 1000 °C. Moreover, it can be observed that as the temperature increased gradually from normal conditions of 80 to 1200 °C, the AAOB and MKBG mixes lost their relative and residual compressive strengths at an accelerated rate. Elevating the ambient temperature has been observed to increase the porosity of the prepared samples, resulting in the generation of secondary porous structures and voids, as well as subsequently reducing the residual compressive strength of both mixtures Figure 3c,d. The detected loss in the mechanical performance is primarily attributed to the mass loss and thermal deformation of the geometrical appearance that are caused by moisture release, as water within the specimen, comprising both free and chemically bound water, moves to the external surface and evaporates, damaging the internal microstructure of the specimen and subsequent strength degradation. This process begins mainly in the temperature range of 100–300 °C,^37^ after that, the rate of strength degradation decreases as the evaporation of water diminishes. Although spalling due to pore pressure reduces at higher temperatures, the residual strength of the prepared samples is affected by the phase transition and changes in composition. At 550–750 °C, a decrease in the compressive strength of both AAOB and MKBG mixtures has been observed which is mainly due to the complete dehydration of chemically bound water present within the Na–Al–Si–H phases, resulting from OB and MK activation process.^38^ The relative compressive strength of AAOB appears to be varied at elevated temperatures, demonstrating a more pronounced sensitivity to change thermal curing conditions compared to that of MKBG specimens, Figure 3e,f.

### 3.2 Weight Loss Rate

**Figure 5:**
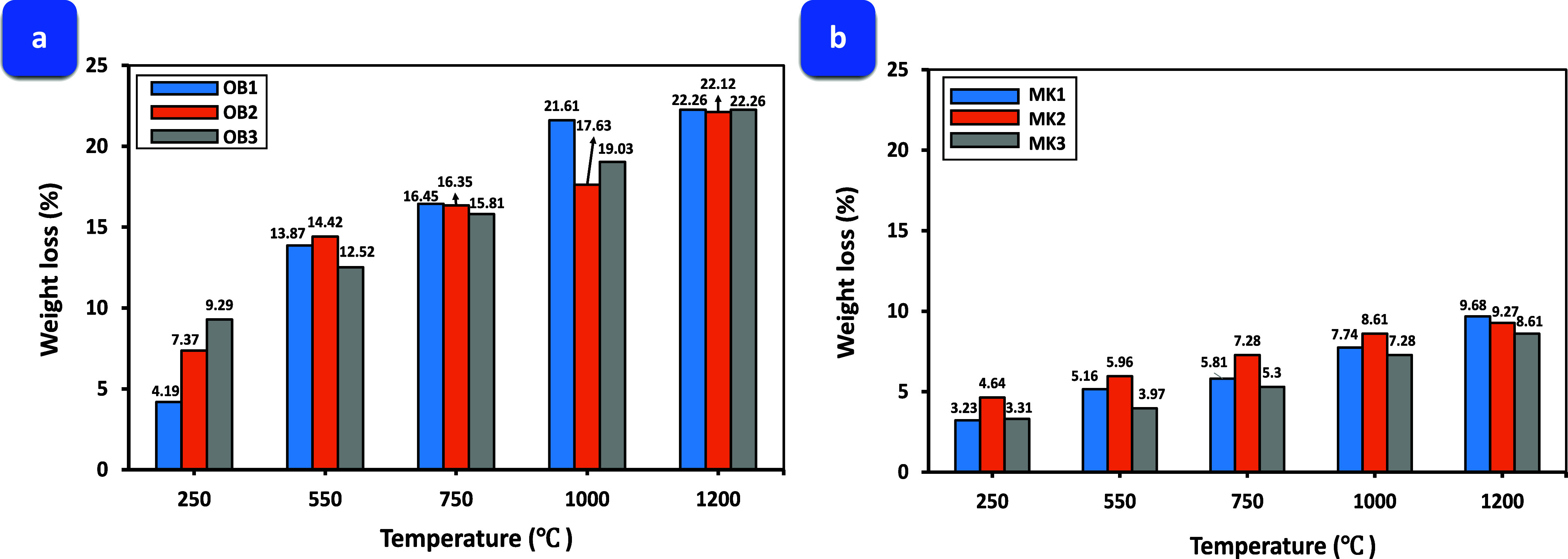
Weight loss rate in (a) AAOB and (b) MKBG samples, respectively.

The mean percentage of weight loss for the AAOB and MKBG samples following exposure to diverse elevated temperatures is displayed in Figure 5a,b. The presented data suggests an apparent correlation between weight loss percentage and elevated temperature, with higher temperatures showing increased weight loss. Notably, increasing the Na_2_O concentration from 8 to 10 and 12 wt % which represented by OB1, OB2, and OB3 obsidian mixtures, respectively, exhibited substantial weight loss percentage increase (4.19, 7.37, and 9.29%, respectively) following exposure to 250 °C. Moreover, the elevation of the exposed temperature from 550 to 1000 °C results in a considerable increase in weight loss across all activated obsidian mixtures. Specifically, the weight loss for OB1 increases from 13.87 to 21.61%, the weight loss for OB2 increases from 14.42 to 17.63%, and the weight loss for OB3 increases from 12.52 to 19.03% up until 1200 °C, where a nearly constant weight loss value is observed for all these samples. This trend was accompanied by a decrease in the compressive strength of all obsidian samples due to the decomposition of all hydrated phases formed during the alkali-activation process of obsidian rock (Figure 5a).

In contrast, MKBG mixtures exhibited lower weight loss values than AAOB samples when subjected to identical elevated temperatures at the same Na_2_O concentrations (Figure 5b). This can be attributed to the high alumina (Al_2_O_3_) content in MK, which acts as a high-temperature-resistant material.^39,40^ The weight loss of all these mixtures is primarily associated with the evaporation of free, adsorbed, and chemically bound water, with the decomposition of hydrated and anhydrate compounds beyond 400 °C also contributing to the weight loss.^31^ This elucidates the observed high weight loss across the mixture samples exposed to temperatures of 1200 °C as compared to those heated at lower temperatures. It is observed that MKBG specimens showed less weight loss than AAOB, this can be ascribed to the curtailed breakdown of hydration products ensuing from the alkali activation of MK can be traced back to the decreased occurrence of Na–Al–Si–H phases, and the reduced reactivity of MK relative to OB is evident.

### 3.3 Relative Volume Change

**Figure 6:**
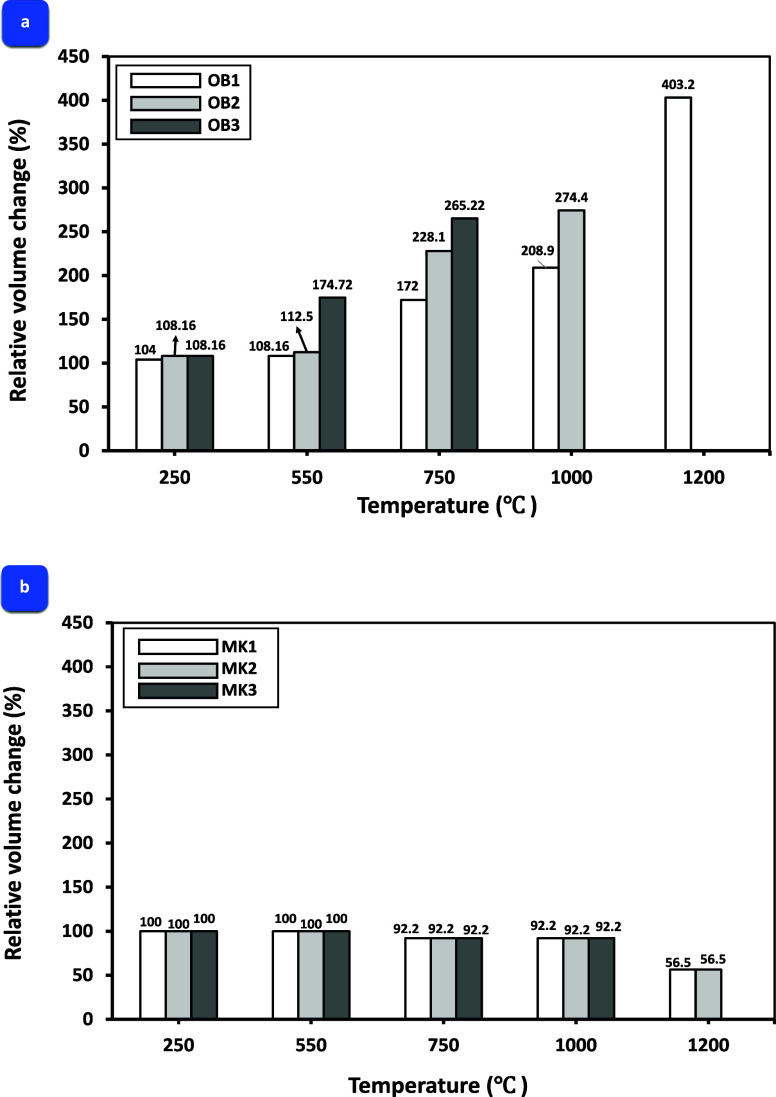
Volume changes in (a) AAOB as opposed to (b) MKBG samples, which show the deterioration of some samples at high temperatures.

The volumetric variations of the AAOB and MKBG samples subjected to various elevated temperatures are presented in Figure 6a,b. The observed changes were induced by the resulting deteriorations such as fractures and/or pores (weak zones), patchy areas, and microscopic cracks. These weak points were formed during the dihydroxylation reaction of Na–Al–Si–H phases resulting from exposure to high temperatures as well as the removal of free and adsorbed water. For AAOB samples, for the mixture with relatively low NaOH content, denoted as OB1, a minimal volume change was observed, in relation to the other samples (OB2 and OB3) at all firing temperatures. This can be attributed to the minimal chemical water removal due to weak Na–Al–Si–H production compared with OB2 and OB3. This finding highlights the influence of the NaOH content on the observed transformations in the samples. Furthermore, the AAOB samples labeled as OB2 and OB3 exhibited more significant growth in volume change due to increasing the chemical water extraction from the internal structure of the developed mixtures, which improved the formation of Na–Al–Si–H at high NaOH concentrations. It is detected that, subjecting the AAOB samples to firing temperatures above 600 °C, there is a quick decline in mechanical properties and a nearly complete loss of compressive strength due to the decomposition of all strength-giving phases.^38^ In addition, the rising temperature to 1200 °C led to the degradation of specific specimens. Notably, OB1 still exists without deterioration up to 1200 °C, while OB2 and OB experienced a decline at the same temperature (Figure 6a).

**Figure 7:**
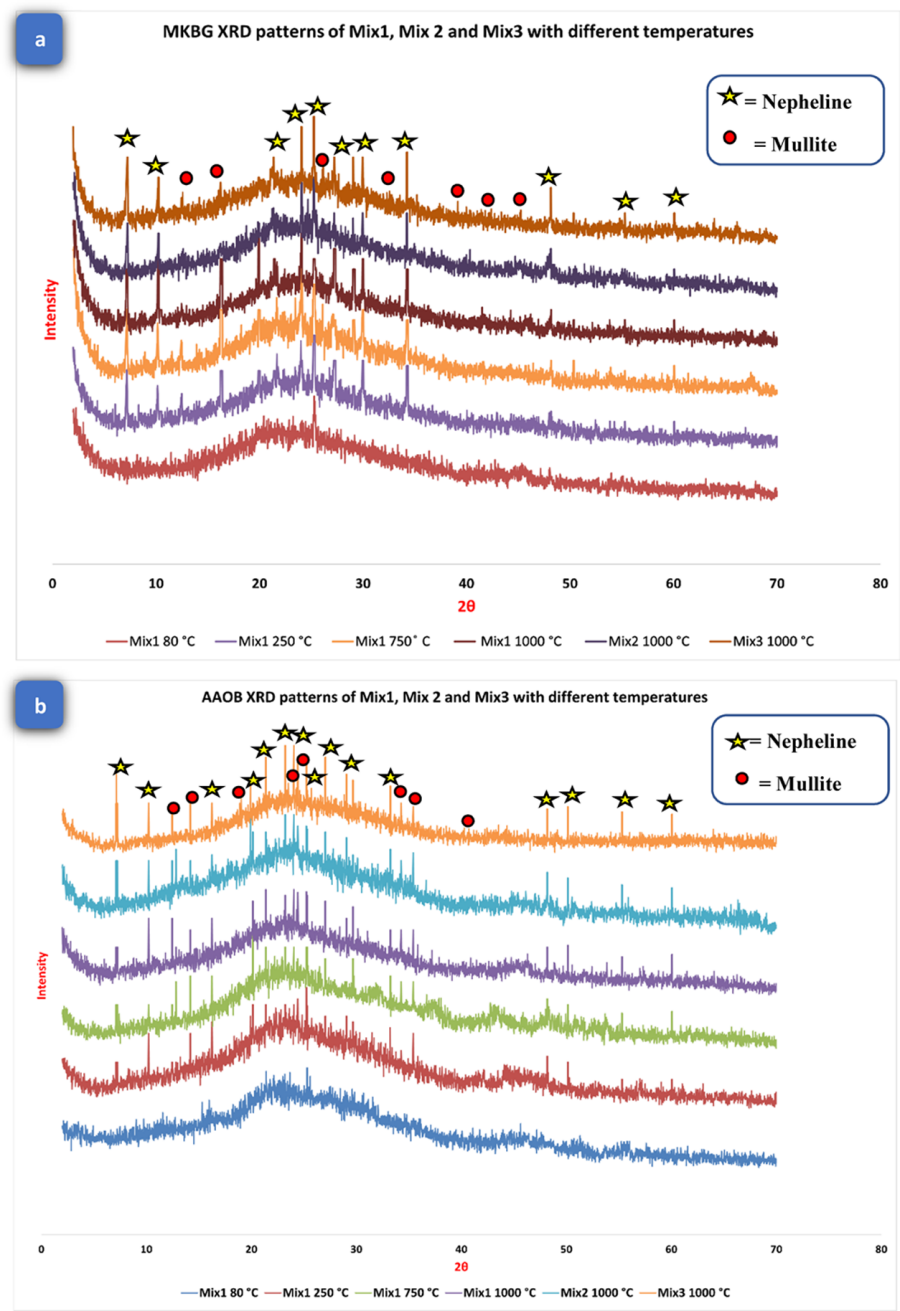
XRD pattern evolution, as a function of temperature, showing changes of (a) MKBG and (b) AAOB samples.

On the contrary, the MKBG specimens did not exhibit any volumetric change when samples were exposed to firing temperatures, spanning 250 to 550 °C. This is ascribed to its high alumina content resulting in high thermal stability.^40^ However, elevating the ambient temperature from 550 to 1000 °C precipitated significant shrinkage in said specimens (92.2%). This can be attributed to the limited decomposition of hydration products resulting from the alkali activation of MK being influenced by the diminished occurrence of Na–Al–Si–H phases (Figure 6b). Remarkably, firing at high temperatures (1200 °C) resulted in the ultimate deterioration of the MK3 specimen, and significant shrinkage percentages (56.5%) were observed in the MK1 and MK2 specimens which were attributable to the total release of adsorbed and chemically bound water,^41^ and the formation of new phases as it was confirmed below by XRD (Figure 7).

### 3.4 X-ray Diffraction (XRD)

The XRD patterns of MKBG and AAOB samples are depicted in Figure 7a,b, respectively. An amorphous geopolymer gel (Na–Al–Si–H) structure can be detected at 2θ range of 18–40°,^42^ which was observed at both AAOB and MKBG mixtures at 80 °C. Increasing the firing temperature of AAOB and MKBG samples presented significant changes in the developed structures, as well as lowering the viscous sintering temperatures of the established specimens while increasing liquid phase formation. It encourages the crystallization of some ceramic phases, increasing the primary crystalline phases’ diffraction peak intensities.^42^ Mullite minerals have been found in both MKBG and AAOB specimens as well by the crystallization of some alumina-silicate liquid phases at elevated temperatures. Mullite is an uncommon silicate mineral formed when clay minerals undergo contact metamorphism. Al_2_O_3_·2SiO_2_ and 2Al_2_O_3_·SiO_2_ are the two stoichiometric forms that mullite may be transformed.^43,44^ Nepheline, with a chemical formula of (Na,K)AlSiO_4_, has been discovered in all prepared specimens following exposure to fire resistance tests, commencing at 250 °C and escalating to 1000 °C, in parallel with the climbing of the diffraction peaks of mullite phases in both AAOB and MKBG mixtures. The nepheline phase exhibits a distinct significance as a result of the high alkaline content of AAOB and MKBG samples at high temperatures.

Raising the ambient temperatures clearly enhances the diffraction peaks of mullite and nepheline phases at the expense of the early-formed amorphous geopolymer gel. AAOB exhibits a higher proportion of nepheline in comparison to the MKBG, which can be attributed to the greater reactivity of OB than MK (Figure 7b). Remarkably, the partially amorphous geopolymer gel’s broad hump peaks in the range of 18–40° (2θ) have not vanished, proving that the conversion of the gel to ceramics only takes place on the surface of both MKBG and AAOB specimens (Figure 7a). It is advantageous to preserve the fire resistance and heat insulation performance of the geopolymer and alkali-activated blocks for the surface to be transformed into ceramic phases, which maintains the integrity of the entire skeleton structure.^45^

### 3.5 FTIR Spectroscopy

**Figure 8:**
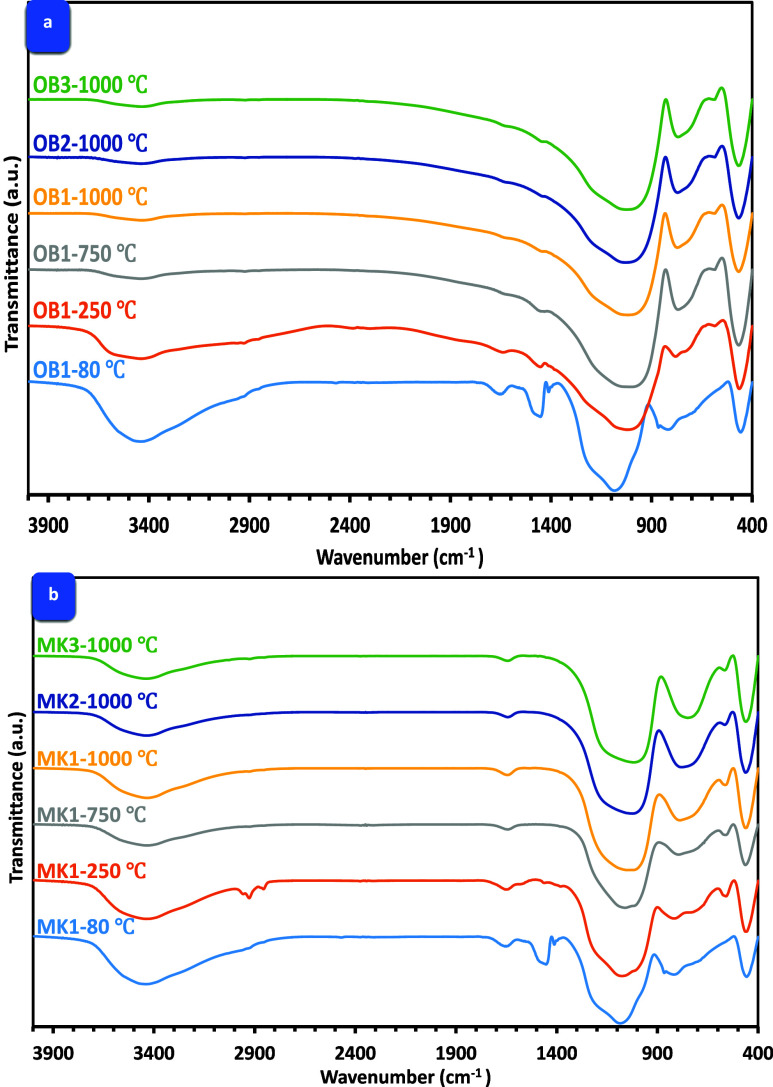
FTIR spectroscopy of (a) AAOB as opposed to (b) MKBG samples.

FTIR spectra of the AAOB and MKBG mixtures at different elevated temperatures are illustrated in Figure 8a,b. Activating OB with NaOH (at Na_2_O weight percent of 8%), the resulting OB1 was observed to exhibit distinct transmittance bands associated with O–Si–O group vibration at 460 cm^–1^, Si–O–Si asymmetric stretching vibration at 1078 cm^–1^^45,46^ and symmetric stretching vibration of the Si–O–Si group at 812 cm^–1^^47^ (Figure 8a). Notably, the spectrum of AAOB exhibited a band at 1454 cm^–1^ which is indicative of the vibration of the C―O bond present in the carbonate group of some existing impurities.^48^ Additionally, the presence of water molecules was confirmed by the observed bending vibration of H–O–H (at 1641 cm^–1^) and the bending vibration of the −OH group (at 3440 cm^–1^).^49^ Upon increasing the firing temperature up to 250 °C, significant shifts and enhancements were observed for the bands of the Si–O–Si group represented at 1078 and 812 to 1000 and 730 cm^–1^, respectively. Such shifts can be attributed to the further polymerization facilitated by the increasing temperature, which results in a higher degree of Na–Al–Si–H cross-linking formation.^50^ Another improvement in the aforementioned silicate peaks was distinguished at 750 °C; this could be affiliated with the crystallization of nepheline and mullite as confirmed by XRD analysis. It was an indication of the AAOB’s ability to withstand high temperatures. Increments in elevation temperature up to 1000 °C lead to a decrease in the intensity of transmittance bands of silicate (1000 and 730 cm^–1^), which is correlated with the cohesionless of the AAOB phases and reducing their compressive strength. Comparable results were obtained in AAOB mixtures activated at different Na_2_O concentrations (10 and 12 wt %).

The FTIR spectra of MKBG exhibit a trio of broad features, with peaks observed at approximately 1080, 800, and 460 cm^–1^ (Figure 8b). The principal intense feature situated at 1080 cm^–1^ has been attributed to the stretching of Si–O bonds present in the amorphous structure of metakaolin. The broad peak appearing at approximately 800 cm^–1^ is assigned to the vibrations of AlO4 tetrahedral units in metakaolin, while the low-frequency signal observed at 465 cm^–1^ is believed to be associated with the Al–O–Si bridges of aluminosilicates.^51,52^ The firing temperature, up to 250 °C, has had a noticeable enhancing effect on the bands of the Si–O–Si group that are represented at 1080 and 800 cm^–1^ and causing a small shift. This can be attributed to an increase in the polymerization process for the hydration products,^53^ as represented in AAOB mixtures. A higher firing temperature of 750 °C leads to a reduction in the peak intensities of Si–O–Si groups in MKBG specimens. This observation suggests that the capacity of MKBG mixtures to endure high temperatures is lower in comparison to that of AAOB. Similar results were found with the other MKBG mixtures under the same conditions.

### 3.6 Porosity and Thermal Conductivity

#### 3.6.1 Porosity

**Figure 9:**
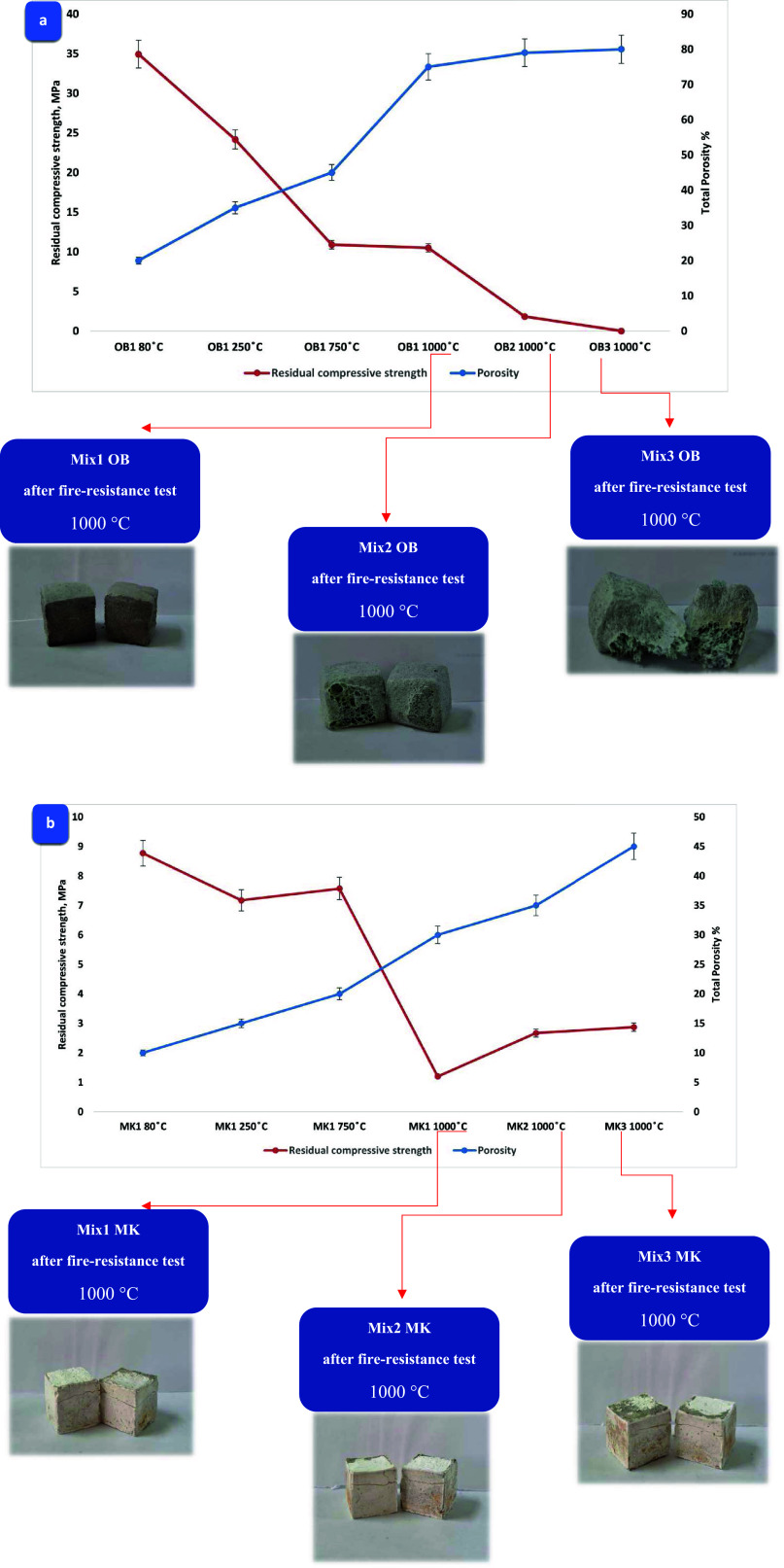
(a) AAOB and (b) MKBG mixtures showing the porosity evolution versus residual compressive strength with varying temperatures.

Illustrating the impact of overall porosity on residual compressive strength, Figure 9a,b visually conveys the relationship. As the firing temperature increased for both AAOB and MKBG mixtures, there was a notable escalation in secondary structural porosity within the samples. This rise was attributed to an augmentation in internal pores, patches, fractures, and voids, particularly noticeable when the temperature climbed from 80 to 1000 °C. Additionally, the heightened porosity, acting as passageways, facilitated the release of internal vapor pressure generated during the heating process. This release, in turn, contributed to a reduction in residual compressive strength.^41,54^ Interestingly, the MKBG mixtures exhibited lower porosity compared to that of the AAOB mixtures. However, despite this, they displayed a diminished residual compressive strength at elevated temperatures.

The AAOB samples exhibit a fascinating porous nature stemming from a transformative sintering process (Figure 9a). This process effectively eliminates absorbed and chemical water from the internal structure of the Na–Al–Si–H phases, inducing a remarkable bone-drying effect. Consequently, both residual strength and thermal conductivity of the samples are significantly influenced.^54,55^ Intriguingly, the elevation of temperatures from 80 to 1200 °C amplifies the development of the porous structure. However, it is worth noting that the development of AAOB surpasses that of MKBG in its striking progression. This discrepancy arises from the intensified decomposition of hydrated phases within AAOB, resulting in a more pronounced porous structure compared to MKBG.

In a surprising turn, the OB1–1000 °C sample stands out as an exceptional case, displaying a notable combination of nearly 80% increased porosity and significantly lowered thermal conductivity. Impressively, this is achieved while still maintaining an acceptable residual compressive strength of 10.5 MPa (Figure 9a). On the flip side, with the ascent of temperatures, the internal structure of MKBG samples undergoes a transformation, resulting in a higher number of pores, though to a lesser extent compared to AAOB (Figure 9b). This evolution adversely impacts the residual compressive strength of MK1–1000 °C, bringing it down to 1.2 MPa with a porosity of 33%. Consequently, this influences the thermal conductivity of the MKBG samples (Figure 9b). Despite the observed increase in porosity and the subsequent decrease in thermal conductivity in MKBG samples, the AAOB mixtures demonstrate even greater porosity and reduced thermal conductivity at comparable firing temperatures.

#### 3.6.2 Thermal Conductivity

**Figure 10:**
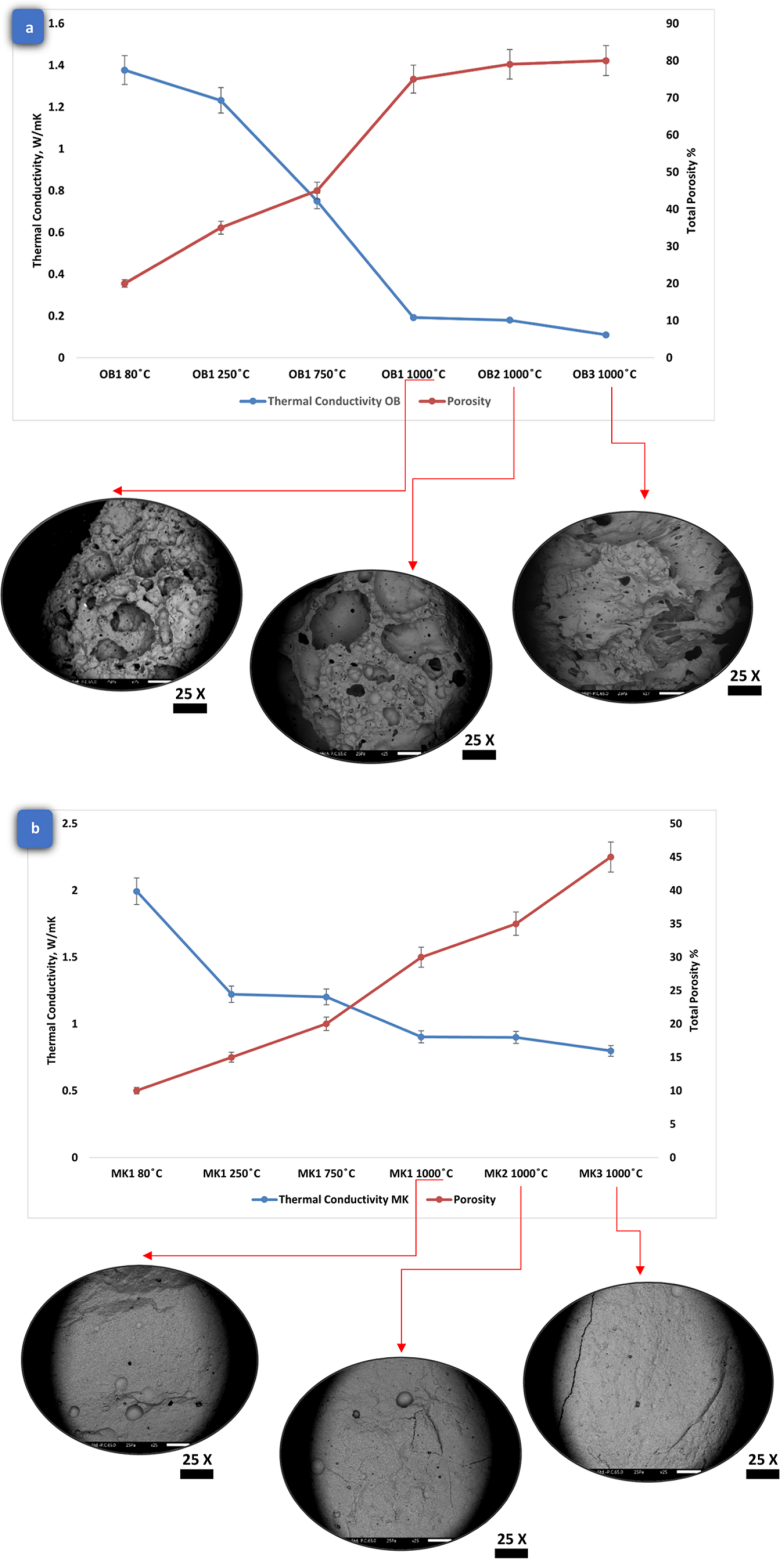
(a) AAOB and (b) MKBG mixtures showing porosity vs thermal conductivity at varied temperatures.

Thermal conductivity was measured with an (Applied Precision ISOMET 2104 analyzer). A slab chip with dimensions of 10*10 mm^2^ length and width respectively with a thickness of 2.3 mm has been prepared. The functionalization of thermal conductivity was achieved through consideration of the overall porosity of the samples, as depicted in Figure 10a,b. The porosity observed in both AAOB and MKBG samples was noted to influence the thermal conductivities, displaying an inverse relationship with porosity. Specifically, at 1000 °C, (OB1) exhibited the highest porosity at 80% and a minimal thermal conductivity of 0.193 W/mK. Conversely, under the same conditions, the MKBG mixture (MK1) showcased the lowest porosity at 33%, accompanied by a maximum thermal conductivity value of 0.902 W/mK Figure 10a,b, respectively.

The highly porous structure was found to act as an effective barrier against heat flow within the geopolymer or alkali-activated network.^54,55^ The porous nature of the AAOB samples resulted from the sintering process that causes the removal of absorbed and chemical water from the internal structure of the Na–Al–Si–H phases. Remarkably, the OB1–1000 °C sample stands out as a noteworthy case, boasting a notable combination of heightened porosity and significantly reduced thermal conductivity with an acceptable residual compressive strength of 10.5 MPa (Figure 10a). On the contrary, MKBG samples show lower porosity and higher percentage of thermal conductivity compared to AAOB mixtures (Figure 10b). In conclusion, despite the noted rise in porosity and the accompanying decline in thermal conductivity in MKBG samples, the AAOB mixtures exhibited higher porosity and lower thermal conductivity at comparable firing temperatures.

### 3.7 TG Analysis

**Figure 11:**
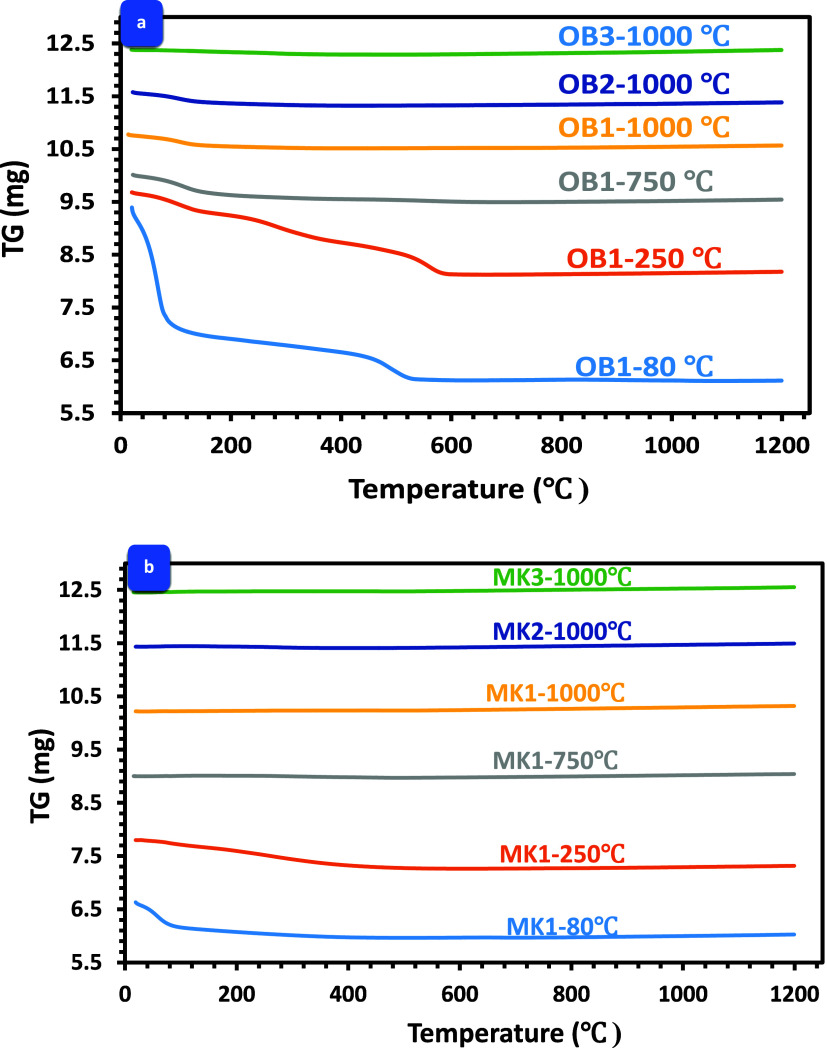
Dynamic TG data of (a) AAOB and (b) MKBG mixtures at different temperatures.

TG analysis of AAOB and MKBG obtained at different temperatures is illustrated in Figure 11a,b. The utilization of NaOH to activate OB at Na_2_O content of 8 wt % (OB1) at 80 °C, resulted in a significant weight reduction (27.4 wt %) between 50 and 160 °C, which can be attributed to the removal of physically adsorbed water linked to Na–Al–Si–H phases,^56^ and unreacted obsidian’s grains. Another weight reduction (8.7 wt %) was observed at temperatures ranging from 200 to 520 °C, caused by the dihydroxylation of chemically bound water present within the Na–Al–Si–H phase.^57^ Increasing the firing temperature of the AAOB mixtures to 250 °C reduced the weight loss resulting from physically adsorbed water and the chemically held water from the Na–Al–Si–H phase; this is due to the elimination of both types of water during the firing process. The aforementioned behavior was observed for all mixtures with the firing temperature raised up to 750 and 1000 °C (Figure 10a).

On the other hand, The MKBG blends exhibited a trend similar to that of the AAOB specimens. Nonetheless, a noticeable distinction was observed in the weight loss of the AAOB mixtures, which exceeded those of the MKBG counterparts (Figure 10b). This effect can be attributed to the greater occurrence of hydration products that were formed in AAOB, owing to the comparatively higher reactivity of OB relative to that of MK.

### 3.8 Scanning Electron Microscopy (SEM) and EDX

**Figure 12:**
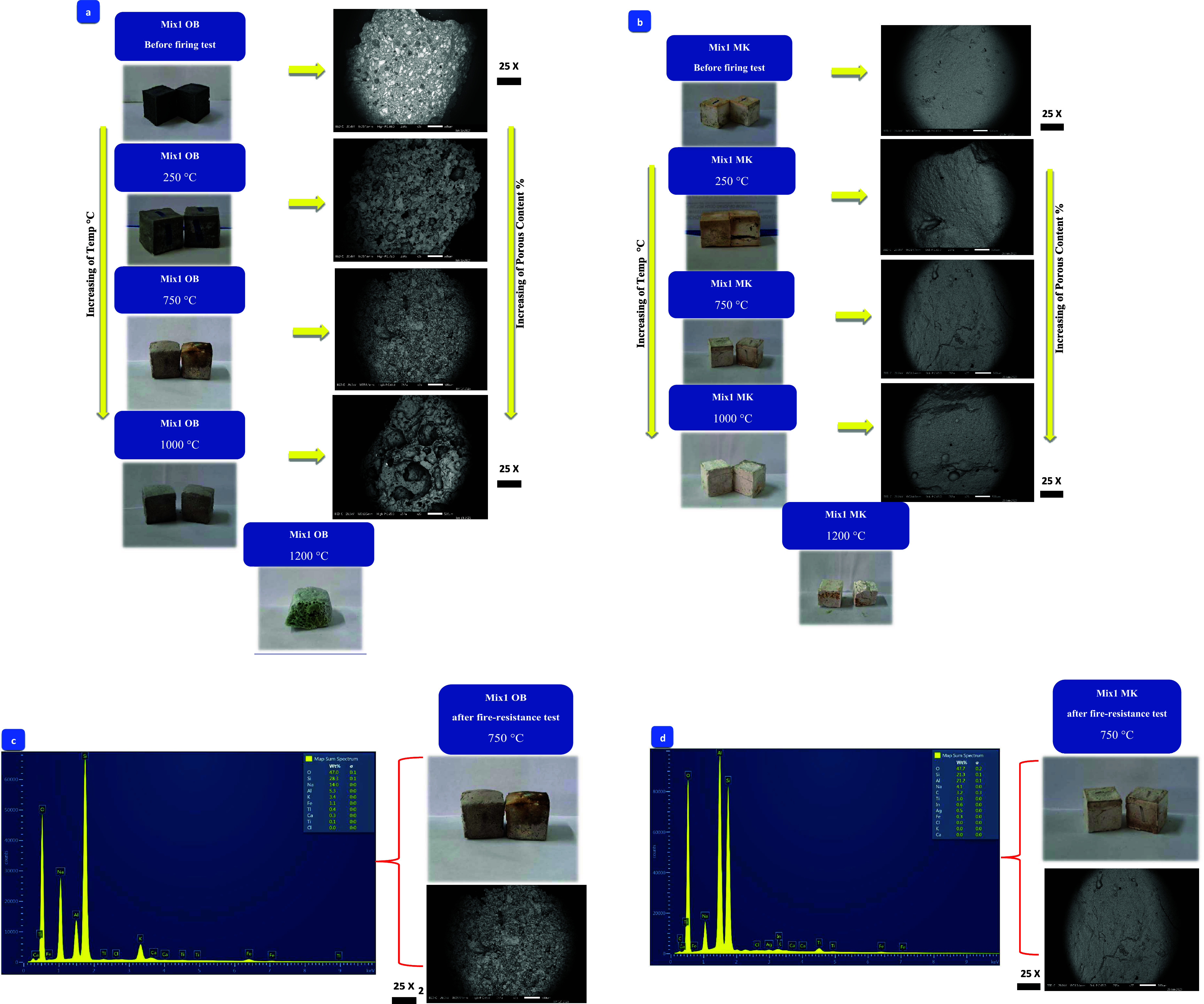
(a, b) Natural images vs SEM of (a) AAOB and (b) MKBG mixtures, as a function of temperature. (c, d) EDX analyses with SEM standard photos showing the percentage compositions of (c) AAOB and (d) MKBG mixtures at a temperature of 750 °C.

The SEM images of both mixtures OB1 and MK1 specimens before and after thermal treatment (Fire exposure test) are displayed in Figure 12a,b. Prior to thermal treatment, neither of the mixtures displayed any observable porous characteristics. The porous texture was revealed through elevating the fire temperature. Noteworthy, SEM images have been opted to showcase captured at a scorching temperature of 1200 °C. Surprisingly, subsequent SEM captures at 250 and 500 °C yielded no discernible distinctions. Regrettably, the endeavor to obtain SEM photographs of the AAOB and MKBG samples posed considerable difficulties, as a few specimens had already started to liquefy at an extreme temperature of 1200 °C. Also, the porous diameter started to be large and difficult to determine by SEM even with low magnification scale. The higher the firing temperature rises, the more porous (foaming texture) the produced product was. SEM images showed that the percentage of porous content is higher in AAOB mixtures compared to the MKBG mixtures, lending support to this observation as presented previously in porosity and thermal conductivity (Figures 9 and 10). OB1 porosity content began to develop at lower temperature and rapidly increased with high amounts and broad diameter upon raising the temperatures. It resulted from the sintering process that causes the removal of chemical water from the internal structure of the Na–Al–Si–H phases (Figure 12a). On the other hand, MK1 did not exhibit any porosity at lower temperatures and only began to develop porous structures as the ambient temperature began to rise around 550–750 °C. The reduced formation of Na–Al–Si–H phases impacts the limited decomposition of hydration products caused by alkali activation of MK (Figure 12b). Consequently, the amount of porosity in MKBG is significantly lower than that in AAOB specimens. The SEM results align smoothly with the findings from TGA, FTIR, porosity, thermal conductivity, and compressive strength, which confirms the relatively higher reactivity of AAOB compared to that of MKBG.

#### 3.8.1 Energy-Dispersive X-ray Spectroscopy (EDX)

Figure 12c,d depicts the EDX analysis for both OB1 and MK1 mixtures. Despite their equal activation through Na_2_O (8 wt %), AAOB displays a heightened quantity of hydration phases compared to MKBG, owing to the augmented dissolution of amorphous aluminum silicates within AAOB, in contrast to MKBG at the same firing condition, 750 °C (Figure 12c). Notably, the abundance of Na–Al–Si–H within AAOB surpasses that in the MKBG (Figure 12d). This assertion finds validation in the previous TG, porosity, thermal conductivity, and compressive strength analyses conducted under identical conditions on both AAOB and MKBG samples. This lends support to the notion that the alkali activation of OB was more potent than that of MK at the same conditions, manifesting in a more comprehensive breakdown of hydrated products and a consequent formation of highly porous phases, which are considerably more prevalent in the AAOB binder than in the MKBG samples.

## 4 Exploring the Environmental Footprint: A Concise Analysis of AAOB and MKBG

Cement and concrete are widely used in construction worldwide, but they contribute approximately 8% to human-induced greenhouse gas emissions.^58^ The calcination of limestone, a key process in cement manufacturing, accounts for almost two-thirds of the total CO_2_ equivalent emissions.^59^ Efforts to reduce these emissions have been challenging due to inherent characteristics of the materials.^60−62^ However, innovative thinkers have developed alternative binders, known as eco-friendly binders, which have the potential to revolutionize the global construction industry. An additional crucial factor that is never overlooked pertains to the expenses associated with grinding within the cement industry. The cement sector offers a range of grinding methods contingent upon the specific material being subjected to grinding. Within the cement production process, approximately 26% of the overall electrical power is dedicated to grinding the raw materials. Throughout this grinding procedure, the energy derived from the rotary burner is consumed.^63^

Alkali-activated materials (AAMs), also known as geopolymers materials (GPMs). These eco-friendly binders offer mechanical qualities that are comparable to, or even better than, traditional cement-based concrete, making them an attractive and viable alternative.^6,64^ Scientific studies have shown that the production of alkali-activated materials (AAMs) and geopolymers (GPMs) significantly reduces CO_2_ equivalent emissions by up to 70% compared to Ordinary Portland cement (OPC) concrete.^65−69^ The primary contributors to the global warming potential (GWP) of AAMs and GPMs are alkali activators such as sodium silicate, sodium hydroxide, potassium silicate, and potassium hydroxide, which account for approximately 60% of emissions.^68,70^ However, the amount of alkali activator used in AAMs and GPMs is relatively small (1–15 wt %), resulting in a minimal amount of CO_2_ emissions compared to the OPC industry. Although there have been advancements in the production techniques of sodium silicate, most environmental data still relies on a seminal study conducted by Fawer et al. in 1999.^71^

AAMs and GPMs have been found to significantly reduce the global warming potential (GWP) compared to traditional OPC concrete. The reduction in GWP ranges from 57% for AAMs based on GBFS to 39% for AAMs based on MK. Surprisingly, activators such as sodium silicate and sodium hydroxide, previously thought to have minimal emissions, can contribute between 13 and 33% of the total CO_2_ equivalent emissions depending on the mix design of AAMs or GPMs. Despite the uncertainties in manufacturing and transportation distances, the GWP of AAMs and GPMs remains lower than that of the OPC concrete mixes. One source of uncertainty is variability in precursor and activator manufacturing techniques. Interestingly, even with differences in transportation distances, the transportation of precursors and activators accounts for less than 20% of the overall environmental consequence uncertainty.^72^

**Figure 13:**
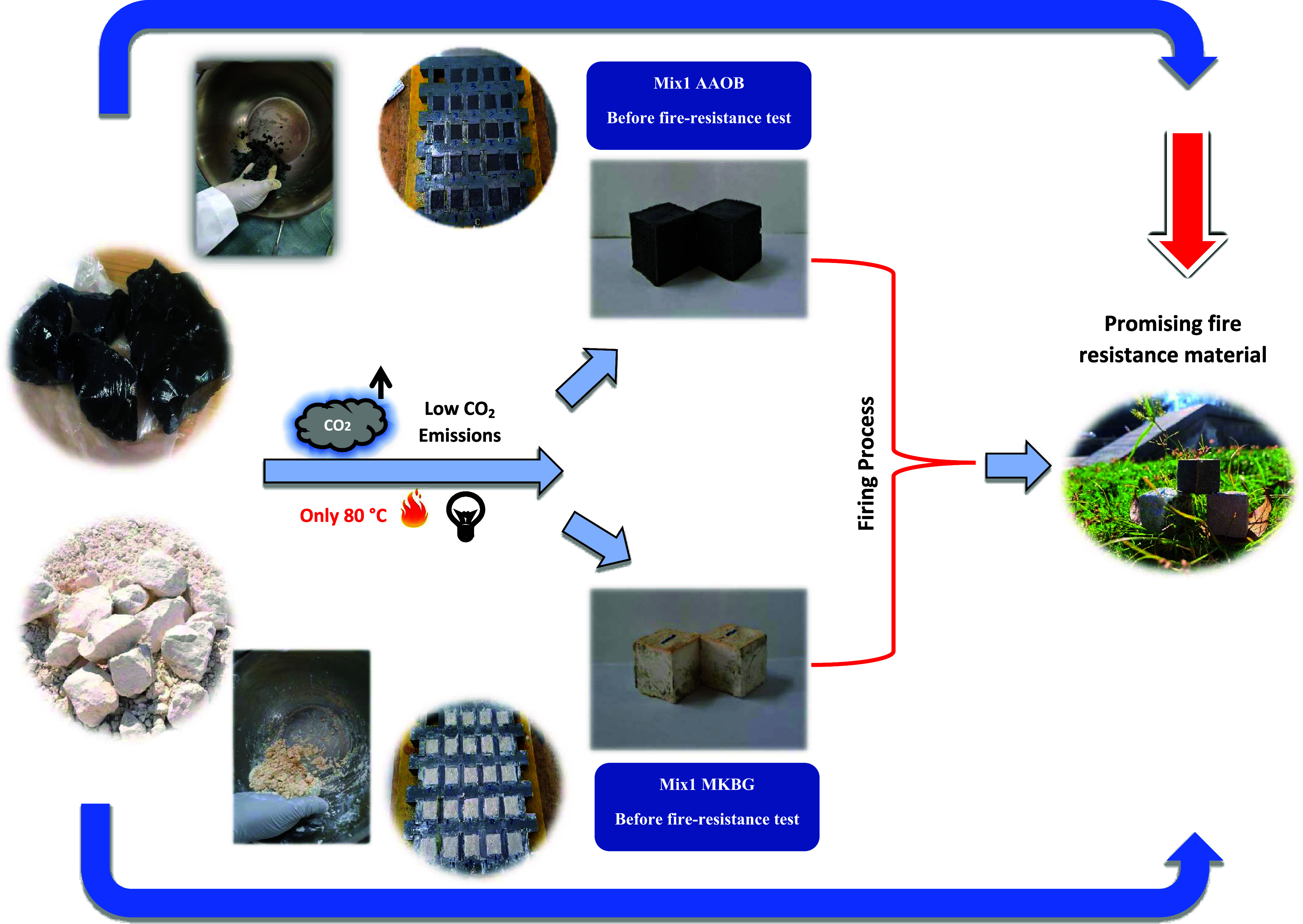
Schematic representation of the whole eco-friendly process (low CO_2_ emissions + low energy consumption) of producing a promising fire resistance for both AAOB and MKBG.

In this study, two remarkable and successful environmentally friendly fire-resistant cementitious materials, namely, MKBG and AAOB have been achieved. What sets these innovative fire-resistant materials apart is their production process, which operates at a relatively low temperature of 80 °C for 24 h, as opposed to the conventional OPC that demands a staggering 1450 °C for manufacturing.^73,74^ The ecological scientific standpoint adopted in this study is illustrated in Figure 13. This method, with its low CO_2_ emissions, diverges from the traditional use of OPC, a notorious contributor to global warming, by embracing AAOB as a promising fire resistance and construction material across numerous applications. This breakthrough significantly reduces energy consumption and CO_2_ emissions, distinguishing it from conventional high-temperature fire-resistant materials.^75,76^

While MKBG undoubtedly possesses its merits, particularly in the realms of high-temperature resistance and thermal conductivity, AAOB possesses the best characteristics. Generally, the natural resources of OB, abundant in Mexico, hold immense potential, requiring no thermal activation as MK does. OB can be utilized in its natural state without requiring thermal activation, unlike MK, which emits a significant amount of CO_2_ during its activation process. Furthermore, the AAOB showcases a notably lower thermal conductivity rate, positioning it as a promising contender for a multitude of applications. Indeed, AAOB stands out as the optimal choice due to its distinct properties. Moreover, this environmentally friendly material exhibits exceptional performance even under the most rigorous conditions, rendering it highly promising for a wide spectrum of applications.

## 5 Conclusions

The study introduced and assessed AAOB, an eco-friendly, sustainable material designed for ultrahigh-temperature applications, excelling in fire resistance and thermal insulation. Synthesized from natural resources, AAOB was produced by blending OB with varying concentrations of NaOH (8, 10, and 12 wt %) and curing at 80 °C. This approach is energy efficient and results in minimal CO_2_ emissions. The same process was used to create MKBG for comparative analysis.

AAOB demonstrated superior performance over MKBG, particularly in mechanical strength, fire resistance, and thermal insulation. Compressive strength tests on the first day showed that AAOB reached 36.5, 69, and 101 MPa, whereas MKBG only achieved 9.1, 23.24, and 25.66 MPa. At 1000 °C, AAOB’s porosity was higher (80%), yet its thermal conductivity was notably lower (0.193 W/mK) compared to MKBG’s 33% porosity and 0.901 W/mK thermal conductivity. Additionally, AAOB maintained a stronger compressive resistance at elevated temperatures. SEM and EDX analyses confirmed these findings, revealing more robust hydration phases (Na–Al–Si–H), attributed to the better dissolution of amorphous aluminum silicates.

The results affirm that AAOB is a cost-effective, low-carbon emission, and efficient thermal insulating cementitious material suitable for industrial and infrastructure use with a minimal global warming footprint. Wide adoption of AAOB could substantially mitigate the environmental impacts tied to traditional materials, presenting a more sustainable option for high-temperature applications.
